# Clinical Pharmacy Initiatives Contribute to the Excellent Efficacy of the Dabrafenib/Trametinib Combination for Iodine-Refractory Thyroid Carcinoma: A Case Report

**DOI:** 10.3390/medicina60071037

**Published:** 2024-06-25

**Authors:** Charlotte Donzé, Fanny Leenhardt, Marie Vinches, Marie-Claude Eberlé, Cyril Fersing

**Affiliations:** 1 Nuclear Medicine Department, Institut Régional du Cancer de Montpellier (ICM), University of Montpellier, 34298 Montpellier, France; 2 Pharmacy Department, Institut Régional du Cancer de Montpellier (ICM), University of Montpellier, 34298 Montpellier, France; 3 Institut de Recherche en Cancérologie de Montpellier (IRCM), INSERM U1194, University of Montpellier, Institut Régional du Cancer de Montpellier (ICM), 34090 Montpellier, France; 4 Medical Oncology Department, Institut Régional du Cancer de Montpellier (ICM), University of Montpellier, 34298 Montpellier, France; 5 IBMM, Univ Montpellier, CNRS, ENSCM, 34293 Montpellier, France

**Keywords:** papillary thyroid carcinoma, oral anticancer therapy, pharmacist-led consultations, dabrafenib, trametinib

## Abstract

A 76-year-old female patient presented with an iodine-refractory papillary thyroid carcinoma (PTC), diagnosed eight years earlier, with several lymph node recurrences requiring successive surgeries. Fluorodeoxyglucose ([^18^F]FDG) positron emission tomography/computed tomography (PET/CT) imaging revealed a new unresectable loco-regional recurrence. The patient was diagnosed with a somatic BRAF V600E mutation. Therefore, dabrafenib and trametinib combination therapy was introduced and closely monitored by a dedicated multidisciplinary team, involving pharmaceutical consultations. As early as six weeks after treatment initiation, the patient reported multiple adverse events (AEs) to the clinical pharmacy team, who provided advice on resolving AEs or improving tolerance. Close interprofessional collaboration among healthcare workers involved in the care pathway allowed for the identification of the most opportune times for temporary suspension of treatment (four suspensions over seven months) or dose reduction (two reductions over 3.5 months). This resulted in a total treatment duration (one year) longer than the average times reported in the literature. The patient showed a rapid and excellent response to treatment immediately after initiation, culminating in a complete metabolic response assessed by [^18^F]FDG PET/CT imaging at nine months. Twenty-five months after treatment discontinuation, the disease remained controlled. Overall, dabrafenib and trametinib combination could offer excellent outcomes in selected patients with refractory BRAF-mutated PTC, with additional clinical pharmacy initiatives allowing for the optimized management of AEs and prolonged treatment periods.

## 1. Introduction

In 2021, 44,280 new cases of thyroid cancer were reported in the United States [1]. Within this broad group of diseases, papillary thyroid carcinoma (PTC) represents the most common histological subtype [2]. Contrary to rare but aggressive anaplastic thyroid carcinoma (ATC), PTC is associated with good overall prognosis for most patients, with a 5-year survival rate over 90% [3]. Patients diagnosed with thyroid cancer commonly undergo three initial, sequential interventions, including surgery, radioiodine (RAI) therapy, and levothyroxine supplementation. Nevertheless, some iodine-refractory PTCs require alternative approaches. In particular, kinase inhibitors targeting the vascular endothelial growth factor receptor (VEGFR), such as lenvatinib [4] or sorafenib [5], may be considered. Although these oral targeted therapies have shown an improvement in progression-free survival, their lack of selectivity can result in numerous adverse events (AEs), such as hypertension, proteinuria, asthenia, hand–foot skin reaction, diarrhea, and skin rash [6,7]. Consequently, alternative targeted inhibitors with better tolerability and sufficient clinical efficacy have been sought and investigated. As oncogenic BRAF mutations are commonly found in thyroid cancers [8,9], the blockade of the associated signaling pathway (ERK1/2, overactivated by mutation) proved a clear rationale. Particular interest was shown in the association of dabrafenib (BRAF inhibitor) and trametinib (MEK1/2 inhibitor), already known for its benefits in unresectable or metastatic melanoma with BRAFV600 mutation [10,11]. This combination therapy was approved by the US Food and Drug Administration (FDA) in 2018 for use in BRAFV600E-mutated ATC, and several subsequent studies, case series, and case reports tended to confirm the favorable pathological response rates obtained with the dabrafenib/trametinib association [12,13,14,15]. Nevertheless, the safety profile of this combined targeted therapy is still marred by AEs [16], which are rarely serious but can significantly affect patients’ quality of life. Hence, the occurrence of AEs may call for treatment modifications such as temporary suspension, dose reduction, or definitive discontinuation. Three levels of dose adaptation are recommended, with degressive dosing at each level for both drugs (Table 1).

At present, the combination of dabrafenib and trametinib is increasingly used in the management of thyroid cancer [17], alone or before RAI for redifferentiation [18]. However, such oral anticancer treatments are often paused or suspended prematurely because of toxicities, sometimes before a potential complete and sustainable response is achieved. Yet most of these AEs are manageable not only by reducing doses but also by implementing associated clinical pharmacy initiatives. Indeed, multidisciplinary patient care and good patient education have been shown to play a key role in successful oral anticancer treatment [19]. For example, pharmaceutical oncological consultations demonstrated their value in oral antineoplastic agents management in cancer patients, especially through the detection of drug interactions [20,21]. Extensive education of patients on topics such as correct dosing schedules, management of common AEs, and proper handling and storage precautions showed similar benefits [22,23,24,25,26]. Moreover, pharmacist-led consultations on oral targeted therapies also proved their worth in improving the efficacy of experimental drugs when performed at clinical trial inclusion [27]. Overall, such early, concerted interventions in patient management allow for longer treatment periods [28] and, in some cases, lead to impressive pathologic complete responses. Hence, in the present case report, we aim to demonstrate how a close patient-centered collaboration between clinical pharmacists, specialized nurses, and oncologists can optimize the tolerability and duration of combined treatment with dabrafenib and trametinib for the management of thyroid cancer.

## 2. Case Presentation

**Figure 1:**
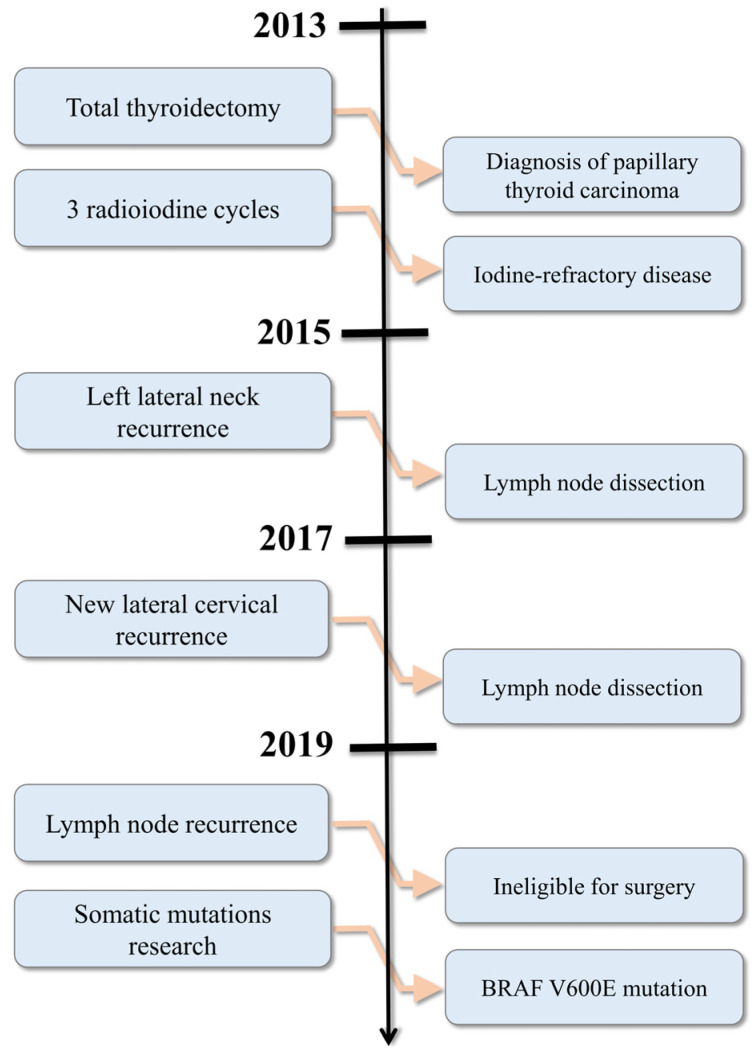
Timeline of patient management before initiation of oral targeted therapies.

A 76-year-old female patient presented to the department of Clinical Oncology (Montpellier Cancer Institute, Montpellier, France) for the follow-up management of a PTC diagnosed eight years earlier. The disease history from diagnosis to initiation of oral targeted therapy is summarized in Figure 1. The patient lived alone, maintained an active lifestyle, had two children, and had an Eastern Cooperative Oncology Group (ECOG) performance status of 0, with a weight of 54 kg and a height of 165 cm (body mass index = 19.8). Her personal history showed no known allergies, a history of appendectomy and cataract surgery, a smoking cessation (evaluated at thirty pack-years), well-controlled hypertension, and chronic obstructive pulmonary disease (COPD). Usual medications included sodium levothyroxine 125 µg (one tablet in the morning), a fixed combination of 300 mg irbesartan and 25 mg hydrochlorothiazide (one tablet in the morning), 64 µg of inhaled budesonide (two doses in the morning), and a fixed combination of 100 µg beclomethasone and 6 µg formoterol by inhalation (one spray in the morning and evening). In January 2013, the patient underwent a total thyroidectomy with central compartment dissection, allowing for the diagnosis of infiltrating papillary thyroid carcinoma of the left lobe, with homolateral nodal extension, stage pT2N1a (TNM 7th edition). After this initial surgery, the patient underwent RAI therapy with a total of three administrations of iodine-131 capsules. Post-therapy scintigraphy did not detect any iodine-avid lesions, indicating that the patient’s disease was refractory to RAI, as structural disease was identified. Indeed, two years after the diagnosis, a first recurrence in the left side of the neck was identified and subsequently treated with a left jugulo-carotid and lower-left spinal lymph node dissection. Four years after the diagnosis, the patient underwent another lower jugulo-carotid lymph node dissection, between the internal jugular and left supraclavicular regions, showing a novel nodal recurrence with extension to the superomedial thymic region. Six years postdiagnosis, a third nodal recurrence was observed in left supra and infra-clavicular region, but this time unresectable.

**Figure 2:**
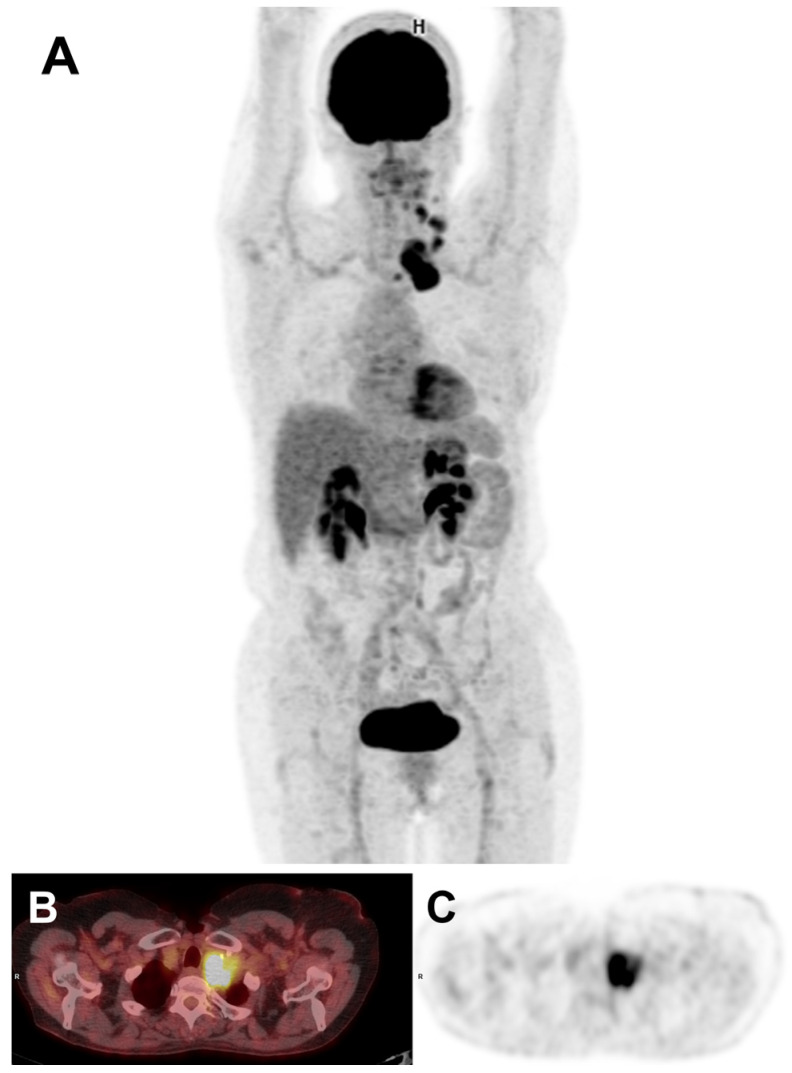
[^18^F]FDG PET/CT imaging showing cervico-thoracic progression of the disease: (**A**) MIP (maximum-intensity projection) images; (**B**) axial fused PET/CT slice; (**C**) axial PET slice.

One year later, a search for somatic mutations in genomic DNA was conducted (French cancer center [INCa] panel, next-generation sequencing [NGS] technique) and revealed a BRAFV600E mutation (located on exon 15, with the genetic alteration c.1799T>A and the protein alteration p.Val600Glu, with an allelic frequency of 6.68%). Subsequent fluorodeoxyglucose ([^18^F]FDG) positron emission tomography/computed tomography (PET/CT) imaging identified a progressive cervico-thoracic evolution of the disease, manifesting as two main tissue masses above and below the left clavicle extending to the pulmonary apex and carotid axis (Figure 2). This was accompanied by left cervical lymphadenopathy in the mid jugulo-carotid and inferior spinal region, making the patient ineligible for further surgery.

## 3. Treatment

After the multidisciplinary board involving oncologists, endocrinologists, and nuclear medicine physicians, a combination of dabrafenib (150 mg twice daily) and trametinib (2 mg once daily) was introduced at the end of July 2020. With the initiation of this treatment, the patient was included in a patient pathway offered by our institution (Montpellier Cancer Institute, Montpellier, France) for oral anticancer treatments, wherein the pharmacist plays a central role (detailed course provided in Section 4 (Discussion)). During the pharmaceutical consultation at treatment initiation, no drug–drug interactions between current treatments and newly prescribed kinase inhibitors were identified. Similarly, the patient was not taking any herbal drugs or vitamin supplements likely to cause interactions. During the interview, the patient was made particularly aware of these risks. The medical consultation at one-month post-treatment showed good tolerance for these targeted therapies, although a weight loss of 3 kg was observed. Treatment was therefore pursued unchanged. A month and a half after starting treatment, the patient contacted the clinical pharmacy team to report several side effects, as follows: anorexia, food disgust, weight loss, gastralgia, and episodes of excessive sweating. Appropriate advice was provided, including weight monitoring and dietary supplements, in cooperation with the medical oncologist. Two months after the start of dual therapy, the patient’s blood test revealed hypokalemia, prompting the initiation of potassium supplementation (potassium chloride 600 mg, two tablets per day for 7 days) and a follow-up blood test one week later. Low-grade hypotension (100 mmHg systolic) was also reported by the patient, leading to the adaptation of the antihypertensive treatment (reduction in the dosage of the irbesartan + HCTZ combination from 300/25 mg to 150/12.5 mg daily). Two and a half months after initiation, given the persistence of excessive sweating and shivering episodes, which impaired quality of life and increased anxiety in the patient, a 1-week therapeutic break was decided to allow for the side effects to resolve. A follow-up examination by [^18^F]FDG PET/CT imaging three months after the start of combined therapy showed a very good response to treatment, albeit partial for the left retro-clavicular lymph node cluster. Notably, no additional lesion appeared.

**Figure 3:**
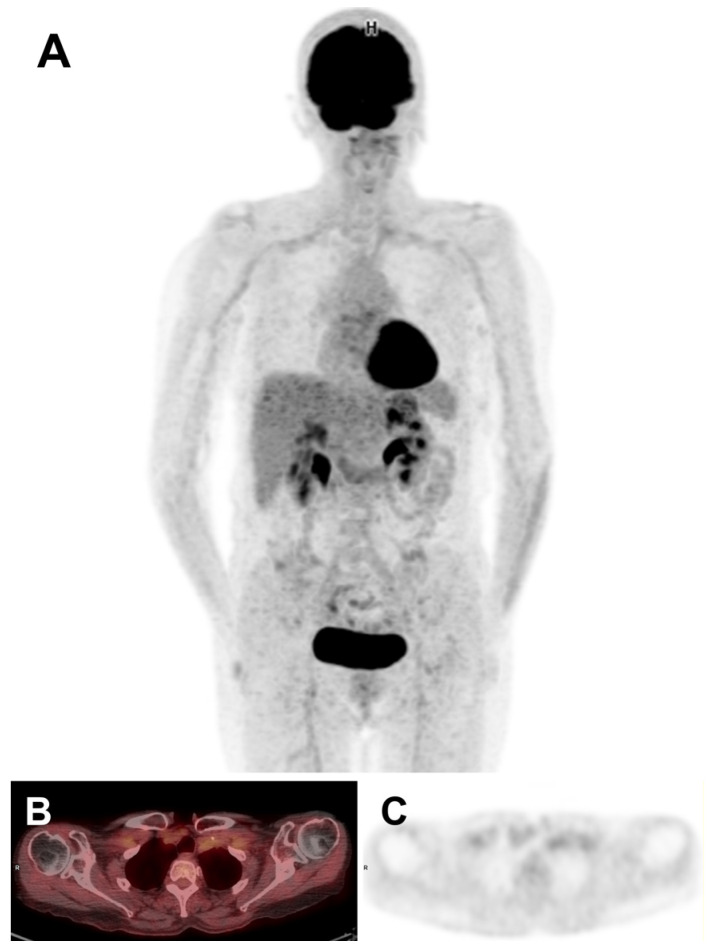
[^18^F]FDG PET/CT imaging showing a complete metabolic response after 9 months of combination therapy: (**A**) MIP; (**B**) axial fused PET/CT slice; (**C**) axial PET slice.

After three months on treatment, tolerance was still poor. Moreover, with the development of high-grade liver toxicity with cholestasis (+300% PAL and +960% GGT in comparison with standard values) and cytolysis (+500% ALAT and +460% ASAT in comparison with standard values), treatment was suspended for a week, until the patient’s clinical course improved and liver function tests normalized. Combination therapy was then resumed at the adapted Tier 1 dose (dabrafenib 100 mg twice daily and trametinib 1.5 mg once daily). Three and a half months after initiation, the patient notified the clinical pharmacists of adverse event recurrence, including low-grade clinical side effects (nocturnal excessive sweating, right leg edema, loss of appetite, and intense cold sensation). In view of these adverse reactions, the patient decided on her own to interrupt the treatment once again. The medical oncologist was immediately informed, and this treatment interruption was formally approved until adverse effects had disappeared. Paracetamol (1 g in the evening) was also prescribed to relieve night sweats. In addition, an echo Doppler was performed to rule out the risk of deep or upper venous thrombosis associated with the leg edema. Lastly, the combination therapy dosing was decreased again, moving up to Tier 2 (dabrafenib 75 mg twice a day and trametinib 1 mg once a day). A few days later, given the persistence of night sweats despite paracetamol, the treatment was suspended again for one week. Four months after starting dual therapy, the patient contacted the clinical pharmacy team to report the reappearance of tremors, weight loss, and night sweats. Furthermore, five and a half months after the start of treatment, the levothyroxine dose had to be increased from 125 µg to 150 µg per day in view of a rise in TSH (1.86 mU/L). Despite these adverse reactions and therapeutic breaks, the six-month follow-up [^18^F]FDG PET/CT imaging showed a further improvement in treatment response, with a reduction in the metabolism of the left clavicular lymph node bundle (78% reduction in SUV compared with the baseline PET). Treatment was therefore maintained unchanged at the appropriate Tier 2 dose. At seven months postintroduction of targeted therapies, a recurrence of low-grade AEs (nocturnal sweating, tremors, anorexia, and nausea) led to a one-week therapeutic interruption, which resolved these toxicities. After the restart of treatment, a further rise in TSH (2.85 mIU/L) prompted a new increase in the levothyroxine dosage (175 µg per day). Table 2 outlines the main AEs reported by the patient during the first 8 months of treatment, for which the clinical pharmacy team was systematically solicited. Remarkably, [^18^F]FDG PET/CT imaging at nine months showed a complete metabolic response (Figure 3).

In view of this favorable outcome and because of small (<1 cm short axis) equivocal persistent lymph nodes on an ultrasonographic examination in the left cervical region, a multidisciplinary discussion on the possibility of redifferentiation was held. Indeed, it has been shown that treatment with dabrafenib and trametinib can increase or restore RAI uptake in tumors, enabling treatment with RAI in initially refractory diseases [29]. This would allow for prolonged suspension of the targeted therapy, which would only be resumed in case of novel progression.

**Table 2:** Summary of AEs experienced by the patient during treatment with dabrafenib plus trametinib. Low grades = 1–2; high grades = 3–4.

Time after Treatment Initiation (Months)	Adverse Event Experienced	Grade (CTCAE v5) [30]	Actions Taken
1	Weight loss (2 kg)	low	Treatment unchanged
1.5	Loss of appetite, weight loss (1 kg), stomach pains, nocturnal excessive sweating	low	Treatment unchanged, dietary and health advice
2	Hypokalemia	low	Potassium supplementation (2 pills per day for 7 days) + biological check-up 1 week later
2.25	Low blood pressure	low	Decrease in dosage of irbesartan from 300 mg per day to 150 mg per day
2.5	Nocturnal excessive sweating, chills	low	Treatment suspension for 1 week
3	Liver toxicity	high	Treatment suspension for 1 month and treatment resumption at appropriate dose → First dose reduction
3.5	Nocturnal excessive sweating, edema of the right leg, loss of appetite	low	Treatment suspension for 1 week Paracetamol 500 mg in the evening Echo-Doppler Treatment resumption at an appropriate dose → second dose reduction
4	Tremors, weight loss, nocturnal excessive sweating	low	Treatment unchanged
5.5	TSH increase	low	Increase in levothyroxine dosage from 125 µg to 150 µg per day
7	Nocturnal excessive sweating, nausea, tremors, loss of appetite	low	Treatment suspension for 1 week
8	TSH increase	low	Increase in levothyroxine dosage from 150 to 175 µg per day

**Figure 4:**
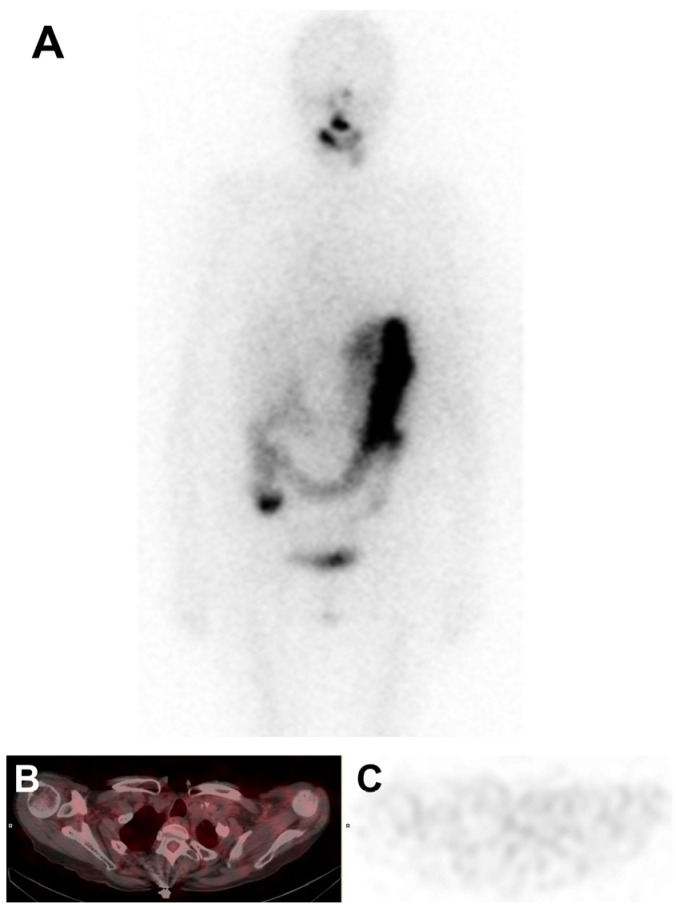
Post-RAI scan showing no abnormal ^131^I uptake: (**A**) maximum-intensity projection (MIP); (**B**) fused axial SPECT/CT slice; (**C**) axial SPECT slice.

Consequently, eleven months after starting combination therapy, the patient received 5.55 GBq of RAI after stimulation with recombinant human thyrotropin (rhTSH). Post-RAI scintigraphy showed no iodine uptake in the left supra- and retro-clavicular lymph node region, as well as in the upper mediastinum (Figure 4). In this context, targeted therapy was definitively discontinued one year after initiation.

**Figure 5:**
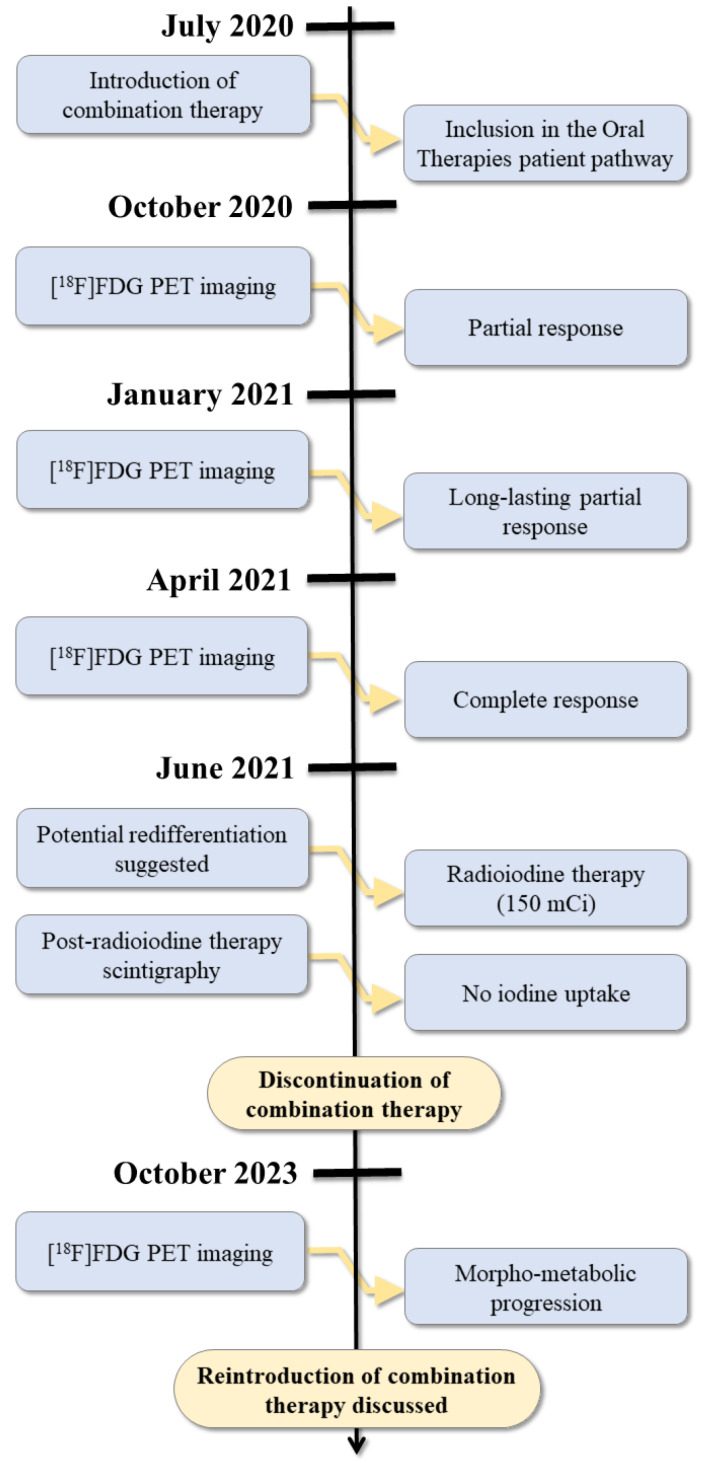
Timeline of patient management from discontinuation of oral targeted therapies to last follow-up visit.

The [^18^F]FDG PET/CT examinations performed one, three, and nine months after discontinuation of the targeted therapy still showed an excellent oncological evolution, with no signs of recurrence, indicating that the disease was in remission. The reappearance of a discrete hypermetabolism focus in the left supraclavicular fossa was identified on [^18^F]FDG PET/CT imaging at twelve months post-treatment and confirmed on [^18^F]FDG PET/CT imaging at fifteen months (SUV max increasing from 3 to 4.9). Close monitoring of the disease was therefore continued. A moderate trend toward morpho-metabolic progression of the left supra-clavicular lymph node infiltrate was found on the follow-up [^18^F]FDG PET/CT imaging performed 25 months after treatment discontinuation. Disease management from the initiation of dabrafenib plus trametinib to post-treatment follow-up is depicted in Figure 5.

## 4. Discussion

Over the last decades, efforts to elucidate the molecular basis of cancers has led to the development of numerous targeted therapeutics for oncology applications, either monoclonal antibodies or small molecules kinases inhibitors [31]. Consequently, a new paradigm has emerged in the management of malignant tumors, guiding therapeutic decisions on the basis of both the molecular signature of the disease and the organ involved [32]. In PTC, predominant modifications occur independently within the MAPK signaling pathway, as approximately 80% of documented alterations in this cancer involve mutant BRAF, RAS, and RET fusions [2]. Accordingly, BRAF alterations such as BRAF V600E play a crucial role in the ligand-independent activation of the MAPK pathway [33], associated with the initiation and progression of thyroid cancer.

**Figure 6:**
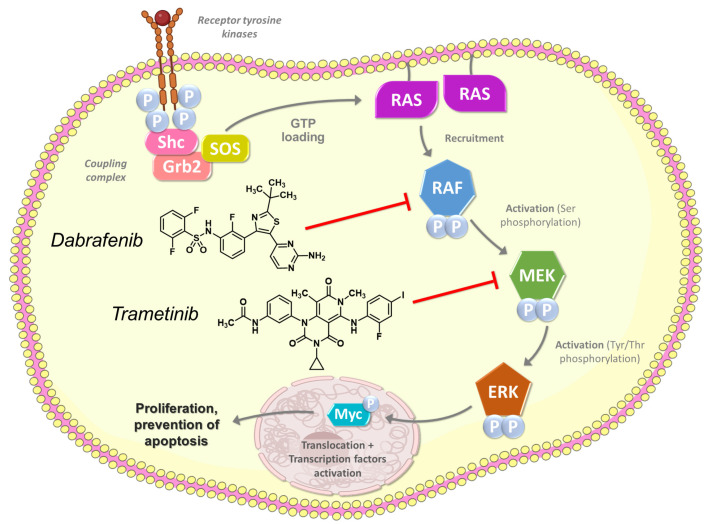
Intracellular MAPK signaling pathway inhibited by the dabrafenib and trametinib combination.

Inhibitors of BRAF and MEK have shown clinical benefit when used in combination, initially in BRAF V600-mutated melanoma for which the following three associations are approved to date by the FDA: vemurafenib/cobimetinib [34,35], dabrafenib/trametinib [10,36,37], and encorafenib/binimetinib [38,39]. In addition, the dabrafenib plus trametinib combination therapy is approved for the treatment of patients with BRAF V600 mutation-positive advanced nonsmall cell lung cancer [40,41,42,43] and showed substantial promise in numerous BRAF-dysregulated solid tumor types [44,45,46,47,48], including thyroid cancers [12,13,29,49]. In such malignancies, dual inhibition of upstream BRAF and downstream MEK can lead to a synergistic effect ending the aberrant oncogenic signal (Figure 6) and could also influence thyroid-specific genes such as those coding for the TSH receptor and the sodium iodide symporter (NIS) [50,51]. Because of these particular effects, the use of the dabrafenib and trametinib combination is a reasonable option for patients with RAI-refractory disease, which can lead to increasing or restoring RAI uptake in tumors through increased NIS expression [29,52,53,54,55]. This so-called redifferentiation approach has also been evaluated with dabrafenib [56,57] or selumetinib [58] (MEK1/2 inhibitor) alone, but regardless of the treatment used, between 40% and 60% of patients do not reach a sufficient redifferentiation level to be successfully retreated by [^131^I]NaI.

In addition to its redifferentiation properties, the combination of dabrafenib and trametinib showed intrinsic efficacy in BRAF-mutated thyroid cancers. For example, Subbiah et al. reported overall response rates (ORRs) up to 56% and a median overall survival of 14.5 months for a median treatment duration of 7 months in patients with ATC [13]. Slightly longer median durations of treatment were reported by Busaidy et al. in patients with differentiated PTC, with a 48% ORR [49]. These studies also highlight that, like many oral targeted therapies [59], the combination of dabrafenib and trametinib can lead to complex toxicities, which could affect patient medication adherence. On the other hand, dose reductions or interruptions are frequently performed in around half of the patients and can improve tolerance and allow for therapy maintenance [13,15,49]. In such a context, integrated, collaborative oral chemotherapy management programs showed a positive impacts on adherence rates, patient understanding of treatment, and overall clinical outcomes [22,23,24,25,26], as in the patient case presented here.

Given the growing use of oral anticancer therapies [60] and the ever-increasing number of marketed agents [61], cancer has become a more chronic disease with care extending over several months or years, calling for an adapted organization in healthcare. In this setting, multidisciplinary consultations (clinician, hospital pharmacist, and specialized nurse) were implemented within our center in 2016 and have recently been integrated into a care pathway for all patients with initial prescription of oral anticancer drugs. The program is based on a multidisciplinary, out-of-hospital follow-up approach, with the objective of strengthening the hospital–community connection. This initiative is based on the following three key objectives: improving patient knowledge of this new therapy, facilitating the management of toxicities, and raising patient awareness of the risks associated with self-medication.

During a patient interview, the nurse reviews the history of the patient’s disease to assess his or her autonomy, identify factors of social vulnerability, evaluate food intake, and investigate possible weight loss. The hospital pharmacist assesses the patient’s understanding of the information provided by the oncologist on the new therapy and can review treatment administration modalities, major side effects, and how to prevent them. To carry out a medication review, the pharmacist discusses with the patient the therapies currently taken. The pharmacist also identifies the patient’s local pharmacy and asks about any herbal products, vitamin supplements, or other additional alternative medicines the patient may use. The nurse–pharmacist team gives the patient prescriptions for the management of potential side effects, along with a treatment summary sheet for the oral anticancer agent, detailing all the information discussed during the interview (see Supplementary Materials). Particular emphasis is placed on the main AEs or disease-related symptoms and how to prevent or treat them if they occur. Possible drug–drug interactions with the oral anticancer agent are investigated, and appropriate pharmaceutical interventions are presented to the oncologist for optimal management. A summary of the interview is then written, archived in the patient’s medical file and sent to the patient’s community pharmacy. A follow-up with the patient over the phone is scheduled after the first week of treatment, during which the nurse assesses the patient’s compliance and treatment tolerance. The nurse will also remind the patient of the importance of reporting any AEs. From this point, further contact is left to the initiative of the patient to report any AEs or ask further question regarding the treatment management to the clinical pharmacy team. This allows optimum reactivity to AEs occurrence and delivery of rapid advice on toxicities management [62]. All calls are reported to the oncology physician and recorded in the patient’s medical file. More than 500 consultations are carried out each year in our center, for more than 40 different oral targeted therapies, enabling patients to take their treatments safely from home. More than 30% of these consultations result in pharmaceutical interventions due to drug–drug interactions between the patient’s medication and oral targeted therapies. The hospital pharmacist is also directly involved in the cancer treatment prescription, proposing dose adjustments of oral targeted therapies in special situations (e.g., drug–drug interactions or renal failure).

Considering the multiple interactions between the clinical pharmacy team and the patient in the present case, the program undeniably played a role in optimizing the management of AEs. This is particularly ensured by telephone contacts following the initial interview [63], which can sometimes take the form of scheduled telephone consultations [28,64]. Toxicity management is a key aspect of the clinical pharmacy services provided to oncology patients [65,66] and can account for a significant proportion of pharmaceutical interventions [67]. A good understanding of the treatment also empowers the patient and facilitates the management of AEs at home, as mentioned in the literature [68]. As a result, it is reasonable to assume that our patient may have benefited from an extended treatment duration supported by the clinical pharmacy actions outlined above. A study involving patients treated with capecitabine showed the benefits of pharmaceutical consultations on the duration of treatment with oral chemotherapy, with a significant increase compared with the control group (83% versus 48% of patients still on treatment at 128 days, *p* = 0.019) [28]. Involvement of the pharmacist in the care process of patients treated with oral anticancer drugs can also be associated with better adherence, either to the treatment itself [67] or to associated measures such as lab parameter monitoring [69]. As previously discussed, such integrated clinical pharmacy services may increase compliance and limit the number of patients who intentionally reduce their oral chemotherapy dose without physician instruction [23]. Finally, our patient was informed about the potential risks of drug–drug interactions, whether caused by prescribed medications or self-administered treatments. Lachuer et al. identified interactions in almost 25% of their patients through the pharmaceutical consultation program they describe [20]. This proportion reaches 43% to 55% in other studies, further underlining the potential key role of the clinical pharmacist in such settings [70,71,72]. Of note, it is essential to consider the possible involvement of complementary alternative medicines (herbal medicines and dietary supplements) in interactions with oral anticancer therapies, as a significant proportion of patients use these products [73,74]. More generally, it was also shown that communications with a clinical pharmacist could reduce anxiety levels in patients undergoing oral chemotherapy [22] and make positive contributions to medical care quality and patients’ quality of life [75,76,77]. The inter-professional collaboration associated with this care process contributes to its efficiency and to a more holistic appreciation of the disease [78,79,80].

Overall, the combination of BRAF inhibitor dabrafenib and MEK inhibitor trametinib can offer excellent outcomes in selected BRAF V600E-mutated thyroid cancer patients, either with ATC or PTC. As demonstrated in the [^18^F]FDG PET/CT imaging, it displayed a complete metabolic response for our patient, despite its substantial AE burden. Remarkably, there was no rebound effect of the disease upon discontinuation of the treatment, with a prolonged response that allowed for a treatment-free interval of 2.5 years before confirmed progression. In this context, the hospital pharmacist initiatives undeniably contributed to the optimized management of AEs related to the treatment and allowed for a prolonged treatment period. Of note, another possibility for improving the tolerability of dabrafenib and trametinib combination in order to achieve or maintain a durable response could be intermittent dosing regimens [81], although the results of this strategy appear inconclusive in melanoma [82]. If adopted, this approach should confirm its relevance through appropriate clinical trials.

This case report has several limitations. The primary limitation is that it focuses on a single patient, which restricts the generalizability of the findings to broader populations with PTC. The follow-up duration, although substantial, might not be sufficient to capture long-term outcomes comprehensively. The use of data in the literature as references, in the absence of a control group, limits the ability to compare effectiveness and safety of the treatment regimen against standard care. Additionally, the subjective nature of some adverse event reports introduces potential bias and variability in assessing side effects. Data on the overall impact of the treatment on the patient’s quality of life are also subjective. Despite these limitations, the report has notable strengths. It provides a detailed account of the patient’s history, treatments, and responses, offering valuable insights for similar cases. The involvement of a multidisciplinary team highlights the crucial role of a collaborative approach in healthcare. The use of dabrafenib and trametinib for treating RAI-refractory PTC showcases an effective therapeutic option. The report underscores the role of clinical pharmacy in managing adverse events, demonstrating the benefits of diligent monitoring and personalized treatment adjustments. Additionally, the discussion on potential redifferentiation therapy offers insights into evolving treatment strategies for PTC.

## 5. Conclusions

A complete and sustained metabolic response was observed after nine months of treatment with dabrafenib plus trametinib in a patient with BRAF-mutated recurrent RAI-refractory PTC. Clinical pharmacy actions, maintained throughout the course of treatment in close collaboration with other healthcare professionals, undoubtedly contributed to optimizing patient care. The benefits of such actions need to be evaluated in patient cohorts compared to control groups and could also be applicable to other types of cancer and oral targeted therapies.

## Figures and Tables

**Table 1:** Recommended dose level reductions for the dabrafenib and trametinib association.

Dose Levels	Dosage of Dabrafenib (Twice a Day)	Dosage of Trametinib (Once a Day)
Initial dose	150 mg	2 mg
Tier 1 dose reduction	100 mg	1.5 mg
Tier 2 dose reduction	75 mg	1 mg
Tier 3 dose reduction	50 mg	1 mg

## Data Availability

The original contributions presented in the study are included in the article/Supplementary Materials, further inquiries can be directed to the corresponding author.

## Supplementary Materials

The following supporting information can be downloaded at: https://www.mdpi.com/article/10.3390/medicina60071037/s1, File S1: Drug information sheet for patients treated with the dabrafenib/trametinib combination, with guidance on managing adverse events.
