# Supplementary material for: Clinical Pharmacy Initiatives Contribute to the Excellent Efficacy of the Dabrafenib/Trametinib Combination for Iodine-Refractory Thyroid Carcinoma: A Case Report
Source: Medicina (Kaunas). 2024 Jun 25;60(7):1037. doi: 10.3390/medicina60071037 (PMC11278742; doi:10.3390/medicina60071037)
Supplement: Supplementary file 1 [file medicina-60-01037-s001.zip › medicina-3053719-supplementary.pdf]

My treatment

......./......./.......

Dabrafenib - Tafinlar® / Trametinib - Mekinist®

50 mg 75 mg 0.5 mg 2 mg

My medical oncologist at the hospital prescribed these treatments.
I can find them in my local community pharmacy under 2 different dosages for both Tafinlar® (50 mg and 75 mg) and Mekinist® (0.5 mg and 2 mg).

Taking my medication

I take my medication according to the instructions on the prescription.

> How?

Tafinlar® twice/day **Mekinist® once/day**

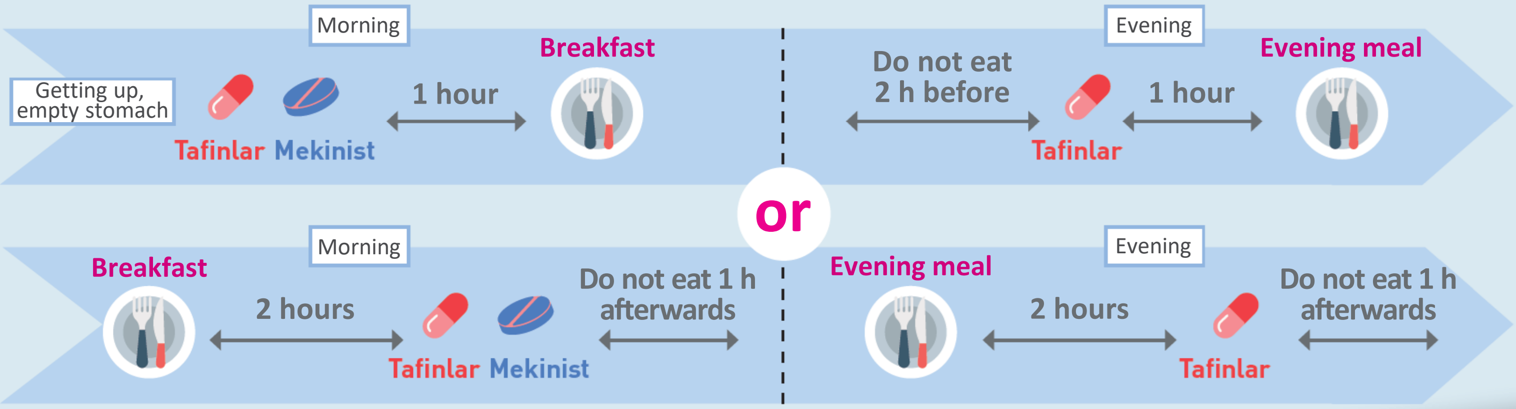

- *Without crushing, breaking or crunching the tablet*
- *Without opening, chewing or melting the capsule*
- *Without ever stopping or modifying my treatment without the approval of my medical oncologist*

> For how long?

I take my treatment every day without a break

Every day

If I have a fever > 38° C, vision disorders or cutaneous rashes, I call my general practitioner or the emergency service, then I inform the clinical pharmacy team

If I vomit after taking my treatment:
- I do not take the treatment again
- I do not double the dose the next time

If I forget to take my medication:
- For **less than 6 hours**: I take the dose
- For **more than 6 hours**: I wait for the next dose

Interactions

If I am taking or wish to take (with or without a medical prescription):
- Medication
- Herbal treatments, essential oils (phytotherapy, aromatherapy), ...
- Certain products (alcohol, smoking, ...)

I inform my medical oncologist or clinical pharmacy team as this may increase side effects and reduce the efficacy of my treatment.

Homeopathy is not concerned.

How to prevent or limit *potential adverse events*

Possible effects, listed by occurrence frequency	What can be done to prevent them	What to do if an adverse reaction occurs
**Nausea / Vomiting** 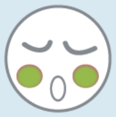	• Eat slowly<br>• Eat several small meals throughout the day<br>• Drink preferably between meals<br>• Avoid fatty, fried and spicy foods	• Favour foods I enjoy<br>• Take the prescribed medication against nausea and vomiting<br>• **Notify my general practitioner if I vomit 3 times in a day, then inform the clinical pharmacy team**
**Diarrhea** 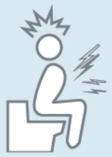	• Avoid coffee, iced drinks, milk, fruit, raw vegetables wholemeal cereals, wholemeal bread<br>• Avoid legumes (lentils, beans beans, peas)<br>• Avoid alcohol	• If possible, drink 1.5 to 2 liters a day: water, herbal teas, bouillons, soft drinks (remove gas first)<br>• Prefer starchy foods (rice, pasta), cooked carrots and bananas<br>• **Notify my general practitioner if I have 4 or more episodes of diarrhea per day, then inform the clinical pharmacy team**
**Skin disorders** (rashes, itching, photosensitivity) 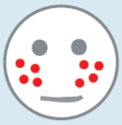	• Use a mild soap<br>• Use a moisturizing cream<br>• Use sunscreen<br>• Avoid prolonged exposure in the sun	• **Notify the clinical pharmacy team as soon as symptoms appear, and then inform my general practitioner**
**Fever, signs of infection** (chills, cough, sore throat, burning sensation during urination) 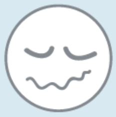	• Take the blood tests prescribed by the medical team<br>• Carefully disinfect any wounds<br>• Limit contact with sick people	• Take my temperature<br>• **Immediately contact** my general practitioner and inform the clinical pharmacy team in case of infection signs: **fever >38°C, chills, sweating, coughing, or burning sensation during urination**
**Fatigue, joint pain** 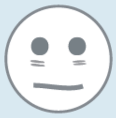	• Take rest periods during the day<br>• Engage in even moderate physical activity	• Being in tune with my body<br>• Be careful when driving a vehicle<br>• **Notify the clinical pharmacy team if my fatigue interferes with my daily activities or persists even with rest**
**Hypertension, edema** 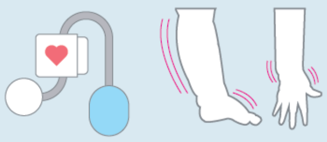	• Take blood pressure readings and record the results for discussion with the doctor<br>• Moderate salt intake<br>• Avoid tight clothing<br>• Monitor weight regularly	• **Notify my general practitioner and inform the clinical pharmacy team in case of:**<br>- **headaches, ear ringing**<br>- **rapid and unexpected weight gain**<br>- **an area of red, inflamed, painful skin**

If an adverse effect persists in intensity or duration, or if other effects not listed appear, I contact: my general practitioner and the clinical pharmacy team, or an emergency service.
